# Rationally Designed Bicyclic Peptides Prevent the Conversion of Aβ42 Assemblies Into Fibrillar Structures

**DOI:** 10.3389/fnins.2021.623097

**Published:** 2021-02-25

**Authors:** Tatsuya Ikenoue, Francesco A. Aprile, Pietro Sormanni, Michele Vendruscolo

**Affiliations:** ^1^Centre for Misfolding Diseases, Department of Chemistry, University of Cambridge, Cambridge, United Kingdom; ^2^Department of Chemistry, Molecular Sciences Research Hub, Imperial College London, London, United Kingdom

**Keywords:** amyloid—beta, Alzheimer’s disease, bicyclic peptides, rational design, protein aggregation

## Abstract

There is great interest in drug discovery programs targeted at the aggregation of the 42-residue form of the amyloid β peptide (Aβ42), since this molecular process is closely associated with Alzheimer’s disease. The use of bicyclic peptides may offer novel opportunities for the effective modification of Aβ42 aggregation and the inhibition of its cytotoxicity, as these compounds combine the molecular recognition ability of antibodies with a relatively small size of about 2 kD. Here, to pursue this approach, we rationally designed a panel of six bicyclic peptides targeting various epitopes along the sequence of Aβ42 to scan its most amyloidogenic region (residues 13–42). Our kinetic analysis and structural studies revealed that at sub-stoichiometric concentrations the designed bicyclic peptides induce a delay in the condensation of Aβ42 and the subsequent transition to a fibrillar state, while at higher concentrations they inhibit such transition. We thus suggest that designed bicyclic peptides can be employed to inhibit amyloid formation by redirecting the aggregation process toward amorphous assemblies.

## Introduction

Since the formation of aberrant deposits composed primarily of the Aβ peptide is a molecular hallmark of Alzheimer’s disease (Selkoe and Hardy, 2016; Jack et al., 2018), a major therapeutic strategy for this condition has been based on the discovery of compounds capable of inhibiting Aβ aggregation (Schenk et al., 1999; Sevigny et al., 2016). However, disease-modifying compounds have not yet become available (Cummings et al., 2020). Major drug discovery efforts have been devoted to the identification of small molecules, which have high brain penetration and low manufacturing costs, but also typically low specificity and high risk of side effects. In parallel, other efforts have been devoted to the development of antibodies, which have the advantage of high specificity, but the disadvantages of high manufacturing costs, difficulty for administration, low permeability, and sometimes poor developability (Sormanni et al., 2018).

**FIGURE 1:**
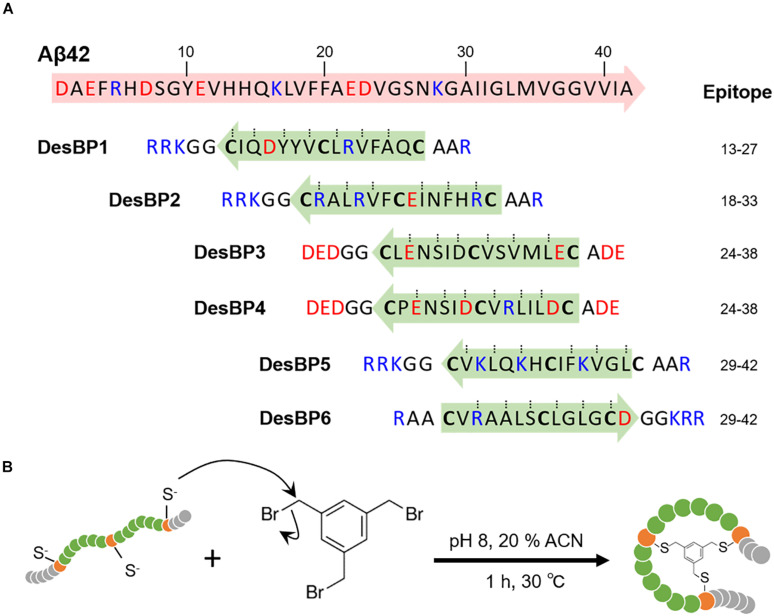
Generation of the rationally designed bicyclic peptides. **(A)** Representation of the six amino acids sequences designed to bind Aβ42 (DesBP1–DesBP6). Three cysteine residues are included for cyclization (bold) and the binding site obtained from the cascade procedure (green and blue arrows) is inserted between cysteine residues. Charged residues are added at N- and C-termini to improve solubility and modulate the binding; positive ones (blue) for DesBP1, DesBP2, DesBP5, and DesBP6, and negative ones (red) for DesBP3 and DesBP4 as controls. Dotted lines mark residues predicted to be involved in backbone–backbone hydrogen bonding and arrows denote the N- to C-termini direction. **(B)** Synthesis of the bicyclic peptides. A rationally designed peptide with three cysteine residues is tethered to the trifunctional compound 1,3,5-tris(bromomethyl)benzene (TBMB) in a nucleophilic substitution reaction.

Bicyclic peptides have recently been introduced in the drug discovery field as they are thought to enable the combination of the advantages of small molecules with those of antibodies (Driggers et al., 2008; Getz et al., 2011; Angelini et al., 2012; Lian et al., 2014; Quartararo et al., 2014; Bartoloni et al., 2015; Bionda and Fasan, 2015). These molecules consist of polypeptide chains where three cysteine residues spaced within the sequence are chemically linked to a cyclic compound. This design results in the formation of two macrocyclic rings that serve as binding regions (Figure 1). As the topology of bicyclic peptides is restrained, they have a relatively small entropy cost upon binding and thus a good binding affinity and specificity (Angelini et al., 2012; Chen et al., 2014; Bionda and Fasan, 2015). Having a small size of about 2 kDa, at least in principle, they are endowed with multiple advantages over antibodies, including the possibility of simple chemical synthesis, better tissue penetration, higher resistance to protease cleavage and inactivation, and extended half-life *in vivo* (Bock et al., 2013).

Bicyclic peptides against specific targets can be developed in a variety of ways. Phage display, for example, can be used for the isolation of these compounds from large combinatorial libraries (Heinis et al., 2009; Angelini et al., 2012; Baeriswyl and Heinis, 2013). This method, however, may become time-consuming and at times ineffective, in particular when one aims at targeting aggregation-prone antigens or weakly immunogenic epitopes. To overcome these limitations, we have introduced a method for the rationally design of antibodies (Aprile et al., 2015, 2017; Sormanni et al., 2015a, 2018) and bicyclic peptides (Ikenoue et al., 2020), which enables the targeting of specific epitopes within intrinsically disordered proteins.

Here, we present an application of this design strategy by generating a panel of bicyclic peptides capable of binding Aβ42 and interfering with its aggregation process. Aβ42 aggregates through a complex process that involves the combination of different microscopic steps and multiple molecular species (Cohen et al., 2013; Michaels et al., 2018). In this context, it is becoming increasingly recognized that the Aβ42 oligomers formed during the aggregation process are highly neurotoxic (Benilova et al., 2012; Mannini et al., 2014). Therefore, therapeutic strategies are being developed to decrease the concentrations of these oligomeric species, for example, by delaying or preventing their formation (Bucciantini et al., 2002; Kayed et al., 2003; Lesne et al., 2006; Haass and Selkoe, 2007; Benilova et al., 2012; Cremades et al., 2012; Cohen et al., 2013; Aprile et al., 2017). In one of such strategies, the amyloid aggregation process is redirected toward off-pathway non-toxic species. The small molecule trodusquemine, for example, can modulate the aggregation process of Aβ42 and by redirecting it toward the formation of off-pathway non-toxic aggregates (Limbocker et al., 2019). Furthermore, strategies aimed at reducing the populations of oligomers by speeding up the aggregation process have also been proposed (Bieschke et al., 2011; Civitelli et al., 2016; Sonzini et al., 2017). Along these lines, we show here that our rationally designed bicyclic peptides prevent the conversion of Aβ42 assemblies into fibrillar structure.

## Results

### Rational Design and Synthesis of Bicyclic Peptides Targeting Different Aβ42 Epitopes

We employed the cascade method, a computational antibody discovery strategy (Sormanni et al., 2015a, 2018; Ikenoue et al., 2020), to generate six bicyclic peptides targeting different regions of the amino acid sequence of Aβ42 (section “Materials and Methods”). These six peptides (DesBP1–DesBP6) were designed to scan epitopes in the most amyloidogenic region of Aβ42 (residues 13–42) (Figure 1A) (section “Materials and Methods”). For the cyclization, we incorporated in the designed sequences three cysteine residues separated by two groups of six residues (Figure 1A). Because the cyclization achieved via reducible disulfide bonds could be problematic for therapeutic purposes, we then used tris-(bromomethyl)benzene (TBMB), a small bromine-containing organic compound, as a scaffold to anchor each designed peptide (Figure 1B). We carried out the reaction in aqueous solvents at 30°C in 1 h, with the threefold rotational symmetry of the TBMB molecule ensuring the formation of a unique structural and spatial isomer. The synthesized bicyclic peptides showed high purity. To assess the solubility of the DesBPs in phosphate buffer, static and dynamic light scattering (DLS) measurements were performed immediately after ultracentrifugation (Supplementary Figures S1a,b). The results showed 50 μM of all the DesBPs remained largely soluble at 5°C, except DesBP4, which formed assemblies of about ∼140 nm in size (Supplementary Figure S1d). Far-UV CD spectra show that DesBP1, DesBP2, DesBP5, and DesBP6 tend to retain structured states (Supplementary Figure S1c). AFM images taken after 1 day, however, showed the presence of assemblies in all cases (Supplementary Figure S1d).

### Characterization of the Effects of the DesBPs on the Aggregation Kinetics of Aβ42

In order to investigate the effects of the DesBPs on Aβ42 aggregation, we carried out *in vitro* aggregation assays using the fluorescent dye thioflavin T (ThT) as amyloid-sensitive probe. We monitored Aβ42 fibril formation at the concentration of 2 μM in the presence of different molar ratios [Aβ42]:[DesBP] (from 0.05 to 16) at 37°C under quiescent conditions, using a highly reproducible aggregation assay previously described (Hellstrand et al., 2010; Ikenoue et al., 2020).

#### Sub-Stoichiometric Concentrations of DesBPs Delay Aβ42 Aggregation and Increase ThT Fluorescence

**FIGURE 2:**
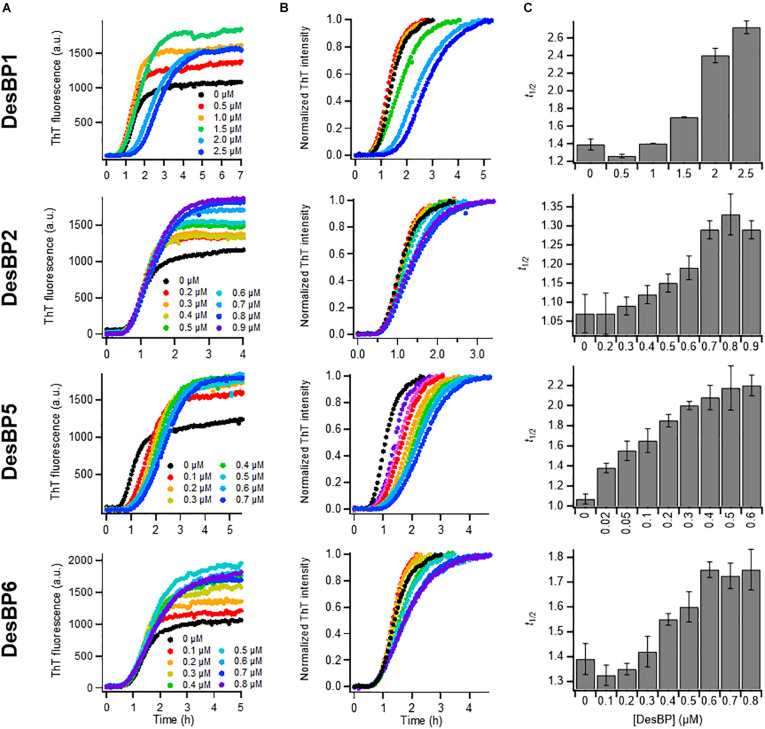
Kinetic analysis of Aβ42 aggregation in the presence of sub-stoichiometric concentrations of DesBPs. Non-normalized **(A)** and normalized **(B)** ThT fluorescence intensity profiles of Aβ42 aggregation under quiescent conditions at a concentration of 2 μM in the presence of increasing concentrations (0.0–0.9 μM) of DesBPs (represented by different colors). Representative curves of three replicates are shown. **(C)** Average half-time (*t*_1__/__2_) of the aggregation reaction at different [Aβ42]:[DesBP] ratios. All experiments were performed in triplicate. Error bars represent standard deviations. For reference, see Ikenoue et al. (2020).

In the presence of low concentrations of DesBPs, we observed significant changes in the ThT fluorescence intensities in the presence of DesBP1, DesBP2, DesBP5, and DesBP6, both in unseeded (Figures 2A–C) and in seeded assays (Supplementary Figure S2), but not in the presence of DesBP3 and DesBP4 (Supplementary Figure S3), a result likely due to the presence of the solubilizing DED motif on DesBP3 and DesBP4. The DED motif generates an electrostatic repulsion with the ED motif on Aβ42, which is likely to interfere with the designed epitope–paratope complementarity (Figure 1A). From the analysis of the normalized curves (Figure 2B), we obtained the dependence of the half-time of aggregation (*t*_1__/__2_) on the concentrations of the DesBPs, which indicate that these bicyclic peptides delay the aggregation process of Aβ42 (Figure 2C).

#### High Concentrations of DesBPs Delay Aβ42 Aggregation and Decrease ThT Fluorescence

**FIGURE 3:**
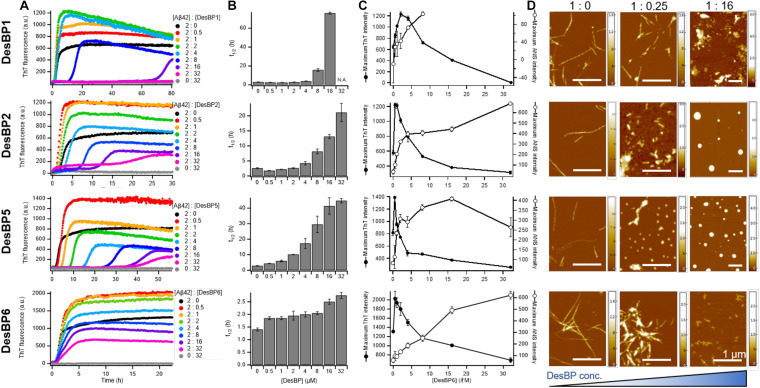
Kinetic analysis and morphological changes of Aβ42 aggregation in the presence of high concentrations of DesBPs. **(A)** ThT kinetic profiles of Aβ42 aggregation under quiescent conditions at a concentration of 2 μM in the absence or in the presence of increasing concentrations (0.5–32 μM) of DesBPs (represented by different colors). Representative profiles of three replicates are shown. **(B)** Average half-time (*t*_1__/__2_) of the aggregation at each [Aβ42]:[DesBP] ratio. **(C)** Averaged maximum ThT (closed circle) and ANS (opened circle) fluorescence intensity at each [Aβ42]:[DesBP] ratio (kinetic profiles are shown in Supplementary Figure S4). All aggregation experiments were performed in triplicate. **(D)** Representative AFM images of Aβ42 aggregates at the end of the ThT assay (32 h) formed in the presence of 0 (left), 0.25 (middle), and 16 (right) molar equivalents of DesBPs. The scale bar on the AFM images indicates 1 μm, and the scale on the right represents the height.

We then tested the effects of high concentrations (0.25- to 16-fold excess) of the DesBPs on the Aβ42 aggregation process (Figure 3). The ThT profiles of DesBP1, DesBP2, DesBP5, and DesBP6 (Figure 3A), but again not of DesBP3 and DesBP4 (Supplementary Figure S3), showed an increase in *t*_1__/__2_ (Figure 3B). At the same time, we observed a suppression of the ThT intensity (Figure 3C) as the concentrations of the DesBPs were increased. To investigate this phenomenon, we studied the morphological changes of the aggregates by using the fluorescent probe ANS, which binds to hydrophobic surfaces. The comparison of the ThT and ANS profiles is shown in Figure 3C, and individual ThT and ANS fluorescence profiles at various concentrations of DesBPs are shown in Supplementary Figure S4. We observed that the ANS intensity was increased in a concentration-dependent manner, while the ThT intensity was suppressed (Figure 3C and Supplementary Figure S4), indicating that the DesBPs induced structural changes to more hydrophobic aggregates.

To further characterize the morphology of the aggregates, we used atomic force microscopy (AFM) after each incubation in the absence and in the presence of 0.25 and 16 molar equivalents of the DesBPs (Figure 3D). Representative AFM images show a morphological transition from fibrillar to non-fibrillar aggregates, consistent with the increase of ANS fluorescence. These different morphologies are presumably caused by the incorporation of the DesBPs into the Aβ42 aggregates. Furthermore, the aggregates did not show seeding ability, apart from those formed in the presence of DesBP6, suggesting that they are not fibrillar (Supplementary Figure S5).

Taken together, our results indicate that these DesBPs extend the lag phase at all concentrations, but appear to exhibit a concentration-dependent mechanism of modulation of Aβ42 aggregation. At low concentrations, the DesBPs increase the ThT intensity (Figure 2), while at higher concentrations, they decrease the ThT intensity by redirecting the aggregation process toward non-fibrillar aggregates (Figure 3).

### Characterization of the Effects of the DesBPs on the Structures of the Aggregates of Aβ42

#### Sub-stoichiometric Concentrations of DesBPs Delay the Aggregation of Aβ42 Into Fibrillar Structures

**FIGURE 4:**
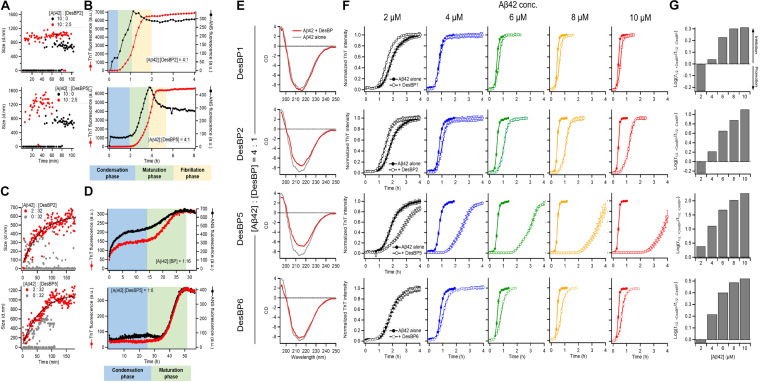
Comparison of the structural properties of Aβ42 aggregates at low and high DesBP concentrations. Kinetics of 10 μM Aβ42 aggregation observed by DLS **(A,C)**, ThT and ANS fluorescence **(B,D)** in the presence of 0.25 and 16 molar equivalents of DesBP2 and DesBP5. Blue, green, and yellow regions in **(B)** and **(D)** represent the condensation, maturation, and fibrillation phases in the aggregation process of Aβ42, respectively. **(E)** Secondary structure of Aβ42 aggregates in the presence of 0.25 molar equivalents DesBPs. Far-UV CD spectra of 10 μM Aβ42 aggregates in the absence (gray) and in the presence (red) of DesBPs. **(F,G)** Kinetics of Aβ42 aggregation in the presence of 0.25 molar equivalents DesBPs for increasing concentrations of Aβ42 (from 2 to 10 μM). Normalized ThT profiles **(F)** (non-normalized ThT profiles are shown in Supplementary Figure S6), and effects of DesBPs on *t*_1__/__2_, the half time of aggregation; the *y*-axis reports the logarithm of the ratio of *t*_1__/__2_ in the presence and absence of a DesBP **(G)**. All experiments were performed in triplicate.

To investigate the structures of the Aβ42 aggregates formed in the presence of the DesBPs, we performed time course DLS measurements to monitor the early stages of aggregation of 10 μM Aβ42 in the presence of 0.25 molar equivalents of DesBP (Figure 4A). The results showed a rapid (within 10 min) appearance of aggregates of about 1.0 μm in size. The ThT profiles, however, did not show any increase until at least 30 min (Figure 4B), showing that these early aggregates do not yet have a fully ordered fibrillar structure. In addition, the growth in the ANS signal within the initial 30 min suggests the presence of hydrophobic assemblies (Figure 4B). Next, in the presence of 0.25 molar equivalents of the DesBPs, the Aβ42 concentration was varied from 2 to 10 μM in the ThT assays. To confirm that the products were still amyloid fibrils, far-UV CD spectrometry measurements were performed in the case of 10 μM of Aβ42. After a 1-day incubation, the CD spectra showed fibrillar structures, although aggregates in the presence of DesBP2 and DesBP5 showed less β-sheet contents (Figure 4E). The ThT profiles indicate that the aggregation of Aβ42 was enhanced in the presence of the DesBPs (Figure 4F), without the formation of significant amounts of off-pathway aggregates (Supplementary Figure S6). We then evaluated the changes the half-time of aggregation, finding that the *t_1__/__2_* values in the presence of DesBP1, DesBP2, DesBP5, and DesBP6 were increased in a concentration-dependent manner (Figure 4G).

#### High Concentrations of DesBPs Delay Aβ42 Aggregation and Promote the Formation of Amorphous Assemblies

In the presence of 16-fold excess DesBP concentration, for 2 μM Aβ42 concentration, the analysis of DLS (Figure 4C), ThT, and ANS (Figure 4D) measurements indicated a gradual formation of amorphous assemblies, presumably of mixed Aβ42/DesBP composition, since the intensity of the ThT and ANS decreases by about 20-fold at high DesBP concentration. At high DesBP concentrations, the aggregates appear to be no longer fibrillar, as also shown by the AFM images in Figure 3 and the seeding experiments in Supplementary Figure S5.

Taken together, these results show that DesBPs promote the condensation of Aβ42 monomers into assemblies formed by interacting Aβ42 and DesBP molecules in the early stages of Aβ42 aggregation. The presence of these assemblies delays, or even blocks, the formation of structured aggregates in the late stages.

## Conclusion

**FIGURE 5:**
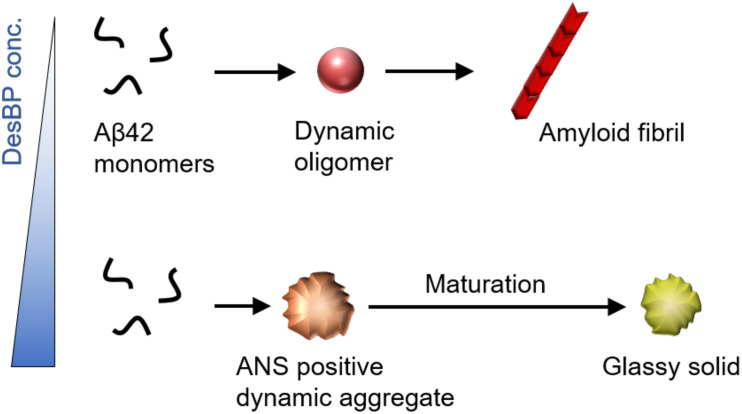
Effects of the DesBPs described in this work on the aggregation process of Aβ42. Our results show a condensation of mixed Aβ42/DesBP assemblies in the early phases of the aggregation process. In the late phases, at low DesBP concentrations, these assemblies are sufficiently dynamic to be able to convert into fibrillar structures, as is generally the case for Aβ42 aggregation (Michaels et al., 2020), while at high DesBP concentrations, they become unable to convert, and mature into amorphous aggregates.

We have described the effects on the aggregation process of Aβ42 of a panel of bicyclic peptides designed to bind different epitopes along the Aβ42 sequence. Our results show that in the early phases of aggregation, there is a condensation of mixed assemblies formed by the Aβ42 and DesBP molecules (Figure 5). In the late phases, at low DesBP concentrations, these assemblies tend to convert into fibrillar structures, while at high DesBP concentrations, they mature into amorphous aggregates (Figure 5). These results indicate that bicyclic peptides can be used to remodel the Aβ42 aggregation process by redirecting it toward non-fibrillar species.

## Materials and Methods

### Reagents

All reagents were purchased from Sigma–Aldrich, excluding ThT UltraPure Grade (ThT ≥ 95%), which was purchased from Eurogentec Ltd.

### Rational Design of the Bicyclic Peptides

In our rational design strategy, we regard a bicyclic peptide sequence as formed by four regions, which are separated by the three cysteine residues required for bicyclization. In this view, we designed the two central regions to enable the binding to the target epitope, as depicted in Figure 1B. By contrast, we retained some motifs (i.e., Ala-Ala at the N-terminus and Gly-Gly at the C-terminus for DesBP1, DesBP2, DesBP5, and DesBP6) of the amino acid sequences of the two terminal regions in order to facilitate the bicyclization reaction. These regions were further endowed with charged residues to enhance the overall solubility of the constructs. We set the length of the binding sites to six or seven residues, following unsuccessful preliminary attempts to carry out the bicyclization reaction with longer sequences, or without the Ala-Ala and Gly-Gly motifs at the termini. The rational designed was performed with the cascade method (Sormanni et al., 2015a) (Figure 1A). The charged residues at the termini were chosen using the CamSol intrinsic solubility score (Sormanni et al., 2015b).

### Recombinant Expression of Aβ42

Aβ42 peptides (MDAEFRHDSGY EVHHQKLVFF AEDVGSNKGA IIGLMVGGVV IA), here called Aβ42, were obtained as described previously by recombinant expression in the *Escherichia coli* BL21 Gold (DE3) strain (Stratagene) (Habchi et al., 2017). The purification procedure was carried out by sonication of *E. coli* cells, dissolution of inclusion bodies in 8 M urea, ion exchange in batch mode on diethylaminoethyl cellulose resin, and lyophilization, followed by further purification using a Superdex 75 HR 26/60 column (GE Healthcare). Eluates were analyzed using SDS-polyacrylamide gel electrophoresis (SDS-PAGE) for the presence of protein products. The fractions containing recombinant Aβ42 were combined, frozen using liquid nitrogen, and lyophilized again.

### Synthesis of the Bicyclic Peptides

The rationally designed linear peptides were purchased from ChinaPeptides. In order to achieve cyclization, the peptides were dissolved in the reaction buffer (20 mM NH_4_HCO_3_, 5 mM EDTA, pH 8.0) at 625 μM. One-quarter volume of 5 mM TBMB in 100% acetonitrile was added to obtain a final concentration of 500 μM peptide and 1 mM TBMB and incubated for 1 h at 30°C. The cyclized peptides were purified by reversed-phase chromatography on a C18 column using H2O/0.08% trifluoroacetic acid (TFA) and acetonitrile/0.08% TFA as solvents, using a GRACE VYDAC C18 (218TP) column 22 × 250 mm. The correct mass was then validated by analytical LC/MS (Xevo).

### ThT and ANS Fluorescence Aggregation Assay

Solutions of monomeric peptides were prepared by dissolving the lyophilized Aβ42 peptide in 6 M GuHCl. The designed bicyclic peptides in their monomeric form were purified from oligomeric species and salt using a Superdex 75 10/300 GL column (GE Healthcare) at a flow rate of 0.5 mL/min, followed by elution in 20 mM sodium phosphate buffer (pH 8) and addition of 200 μM EDTA. The peptide concentration was determined from the absorbance of the integrated peak area using ε280 = 1495l mol^–1^ cm^–1^. The designed bicyclic peptides were dissolved in water and centrifuged at 20°C for 1 h at 435,000 *g* before use. The obtained DesAbs in their monomeric forms were diluted with buffer to the desired concentration and supplemented with 20 μM ThT and 50 μM ANS from a 1 mM stock. ANS experiments were carried out as described previously (Ikenoue et al., 2020). Seeding experiments were performed in the presence of 10% (v/v) preformed fibrils, as described previously (Ikenoue et al., 2020). Preformed fibrils were prepared by the same procedure used with spontaneous fibril formation. All samples were prepared in low-binding Eppendorf tubes on ice using careful pipetting to avoid introduction of air bubbles. Each sample was then pipetted into multiple wells of a 96-well half-area, low-binding polyethylene glycol coating plate (Corning 3881) with a clear bottom, at 80 μL per well. Assays were initiated by placing the 96-well plate at 37°C under quiescent conditions in a plate reader (Fluostar Optima; BMG Labtech). The fluorescence was simultaneously measured through the bottom of the plate with excitation filter at 440 nm for ThT and 380 nm for ANS and emission filter at 480 nm.

### Static and Dynamic Light Scattering

The light scattering measurements were performed on a Zetasizer Nano S instrument (Malvern Instruments, Malvern, United Kingdom) in backscattering mode at 173°. The instrument was equipped with a light source with a wavelength of 633 nm and a Peltier temperature controller at 25°C. Samples were prepared as described above, and 70 μL of them was pipetted into disposal plastic micro cuvette.

### CD Spectroscopy

Far-UV CD spectra of proteins and peptides in soluble and insoluble states were measured with a J-820 spectropolarimeter (Jasco, Japan) using a cell with a light path of 1 mm at each condition. Individual Aβ42 solutions were prepared at 10 μM for CD measurements. The CD signals between 195 and 250 nm were expressed as mean residue ellipticity [θ] (deg cm^2^ dmol^–1^). Temperature regulation was carried out using a PFD-425S Peltier-unit (Jasco, Japan).

### Atomic Force Microscopy

Atomic force microscopy measurements were carried out in air with the sample deposited on functionalized mica. To functionalize the surface, after cleaving, the bare mica substrate was incubated with a 10 μL drop of 0.05% (v/v) APTES [(3-aminopropyl)triethoxysilane, Fluka] in Milli-Q water for 1 min at room temperature, rinsed with Milli-Q water, and then dried by the passage of a gentle flow of gaseous nitrogen. The preparation of the mica AFM samples was made at room temperature by deposition of a 10 μL aliquot of 10 μM solution for 5 min. Then the samples were rinsed with ultrapure water and dried by a gentle flow of nitrogen.

Atomic force microscopy imaging was carried out in intermittent contact mode on a JPK Nanowizard II AFM recorded with AC mode under ambient conditions using an integral gain of 120 Hz, post-gain of 0.008 Hz, and 0.3 Hz line-rate for 4 × 4 μm images. Images flattening and statistical analysis were performed by SPIP (Image metrology) software.

## Data Availability Statement

The original contributions presented in the study are included in the article/Supplementary Material. Further inquiries can be directed to the corresponding author/s.

## Author Contributions

TI, FA, PS, and MV were involved in the design of research. PS designed the peptide. TI performed the experiments. TI, FA, and MV wrote the manuscript. All authors discussed the results and commented on the manuscript.

## Conflict of Interest

The authors declare that the research was conducted in the absence of any commercial or financial relationships that could be construed as a potential conflict of interest.

## Supplementary Material

Supplementary Figure 1 Solubility of the DesBPs in the absence of Aβ42 before and after incubation. Static **(a)** and dynamic **(b)** light scattering of 50 μM DesBP monomers solved in phosphate buffer at 5°C. **(c)** Far-UV CD spectra of 32 μM DesBP monomers. **(d)** Representative AFM images of 32 μM DesBPs after 1 day incubation at 37°C. The scale bar on the AFM images indicates 1 μm, and the scale on the right represents the height.

Supplementary Figure 2 Seeding experiments of Aβ42 amyloid fibrils at sub-stoichiometric concentrations of DesBPs. Non-normalized **(a)** and normalized ThT kinetic profiles **(b)** of Aβ42 aggregation under quiescent conditions at a concentration of 2 μM in the absence or in the presence of various concentrations (0.1–0.9 μM) of DesBPs (represented by different colors). **(c)** Maximum ThT intensity of the aggregation at each [Aβ42]:[DesBP] ratio. All experiments were performed in triplicate.

Supplementary Figure 3 DesBP3 and DesBP4 do not affect significantly Aβ42 aggregation. ThT **(a)** and ANS **(b)** kinetic profiles of Aβ42 aggregation under quiescent conditions at a concentration of 2 μM in the absence or in the presence of various concentration (0.5–32 μM) of DesBPs (represented by different colors). **(c)** Representative AFM images of Aβ42 aggregates in the presence of 16 molar equivalents of DesBPs. The scale bar on the AFM images indicates 1 μm, and the scale on the right represents the height. All aggregation experiments were performed in triplicate.

Supplementary Figure 4 Kinetic profile of ThT and ANS fluorescence of Aβ42 aggregation at various concentrations of DesBPs. ThT (closed circles) and ANS (opened circles) kinetic profiles of Aβ42 aggregation under quiescent conditions at a concentration of 2 μM in the absence or in the presence of various concentrations (0.5–32 μM) of DesBP1, DesBP2, DesBP5, and DesBP6 (represented by different colors). All experiments were performed in triplicate.

Supplementary Figure 5 Seeded aggregation assay of Aβ42 in the presence of high molar equivalents of DesBPs. The aggregates formed in the presence of the DesBPs, with the exception of the case of DesBP6, did not show seeding ability, indicating that they do not have a fibrillar nature. Experiments were performed in triplicate.

Supplementary Figure 6 Kinetics of Aβ42 aggregation in the presence of 0.25 molar equivalents DesBPs. We report the results for increasing concentrations of Aβ42, from 2 to 10 μM. All experiments were performed in triplicate.
