# Supplementary material for: Rationally Designed Bicyclic Peptides Prevent the Conversion of Aβ42 Assemblies Into Fibrillar Structures
Source: Front Neurosci. 2021 Feb 25;15:623097. doi: 10.3389/fnins.2021.623097 (PMC7947257; doi:10.3389/fnins.2021.623097)
Supplement: Supplementary Figure 1 — Solubility of the DesBPs in the absence of Aβ42 before and after incubation. Static (a) and dynamic (b) light scattering of 50 μM DesBP monomers solved in phosphate buffer at 5°C. (c) Far-UV CD spectra of 32 μM DesBP monomers. (d) Representative AFM images of 32 μM DesBPs after 1 day incubation at 37°C. The scale bar on the AFM images indicates 1 μm, and the scale on the right represents the height. [file Data_Sheet_1.docx]

**Supplementary Information**

**
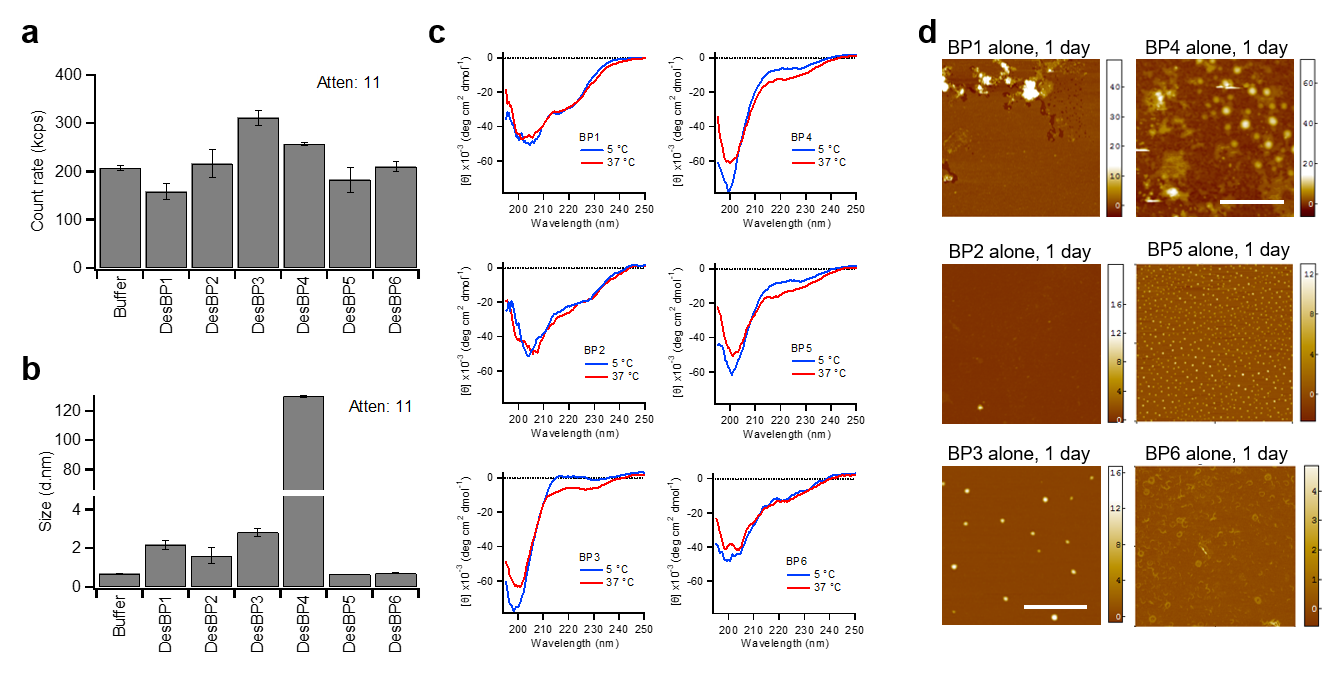
**

**Figure S1. Solubility of the DesBPs in the absence of Aβ42 before and after incubation.** **(a,b)** Static (a) and dynamic (b) light scattering of 50 μM DesBP monomers solved in phosphate buffer at 5 °C. **(c)** Far-UV CD spectra of 32 μM DesBP monomers. **(d)** Representative AFM images of 32 μM DesBPs after 1 day incubation at 37 °C. The scale bar on the AFM images indicates 1 μm, and the scale on the right represents the height.

**
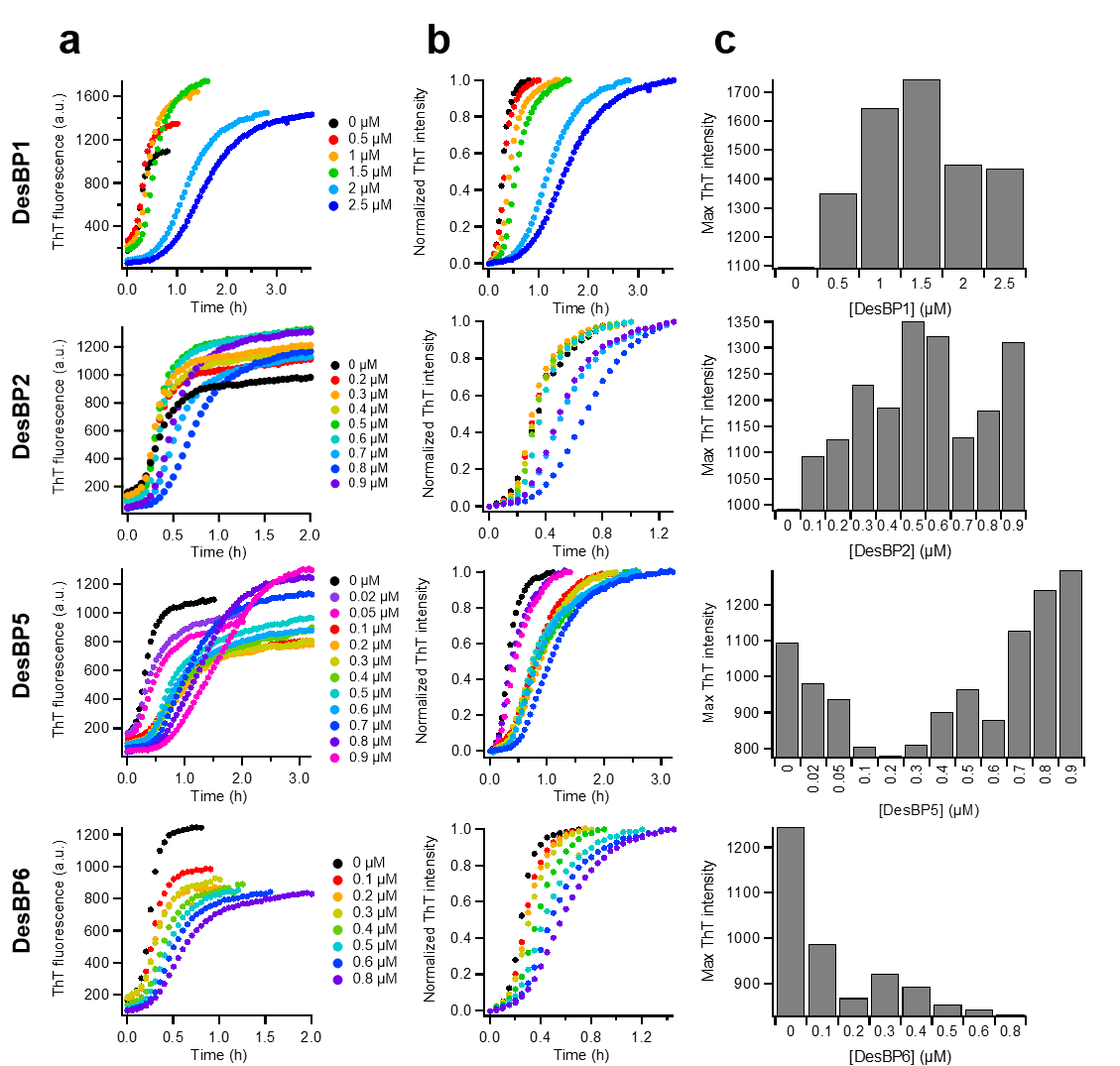
**

**Figure S2. Seeding experiments of Aβ42 amyloid fibrils at sub-stoichiometric concentrations of DesBPs.** **(a,b)** Non-normalised (a) and normalized ThT kinetic profiles (b) of Aβ42 aggregation under quiescent conditions at a concentration of 2 μM in the absence or in the presence of various concentrations (0.1-0.9 μM) of DesBPs (represented by different colors). **(c)** Maximum ThT intensity of the aggregation at each [Aβ42]:[DesBP] ratio. All experiments were performed in triplicate.

**
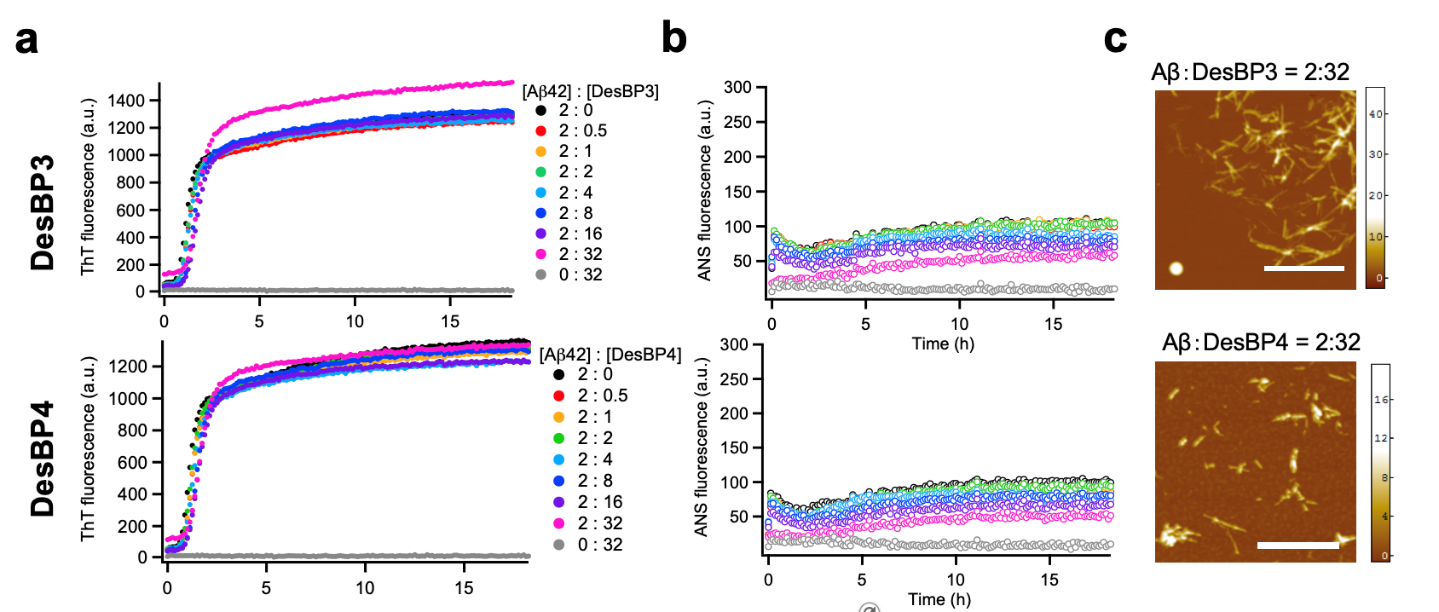
**

**Figure S3. DesBP3 and DesBP4 do not affect significantly Aβ42 aggregation.** **(a,b)** ThT (a) and ANS (b) kinetic profiles of Aβ42 aggregation under quiescent conditions at a concentration of 2 μM in the absence or in the presence of various concentration (0.5-32 μM) of DesBPs (represented by different colors). **(c)** Representative AFM images of Aβ42 aggregates in the presence of 16 molar equivalents of DesBPs. The scale bar on the AFM images indicate 1 μm, and the scale on the right represents the height. All aggregation experiments were performed in triplicate.

**
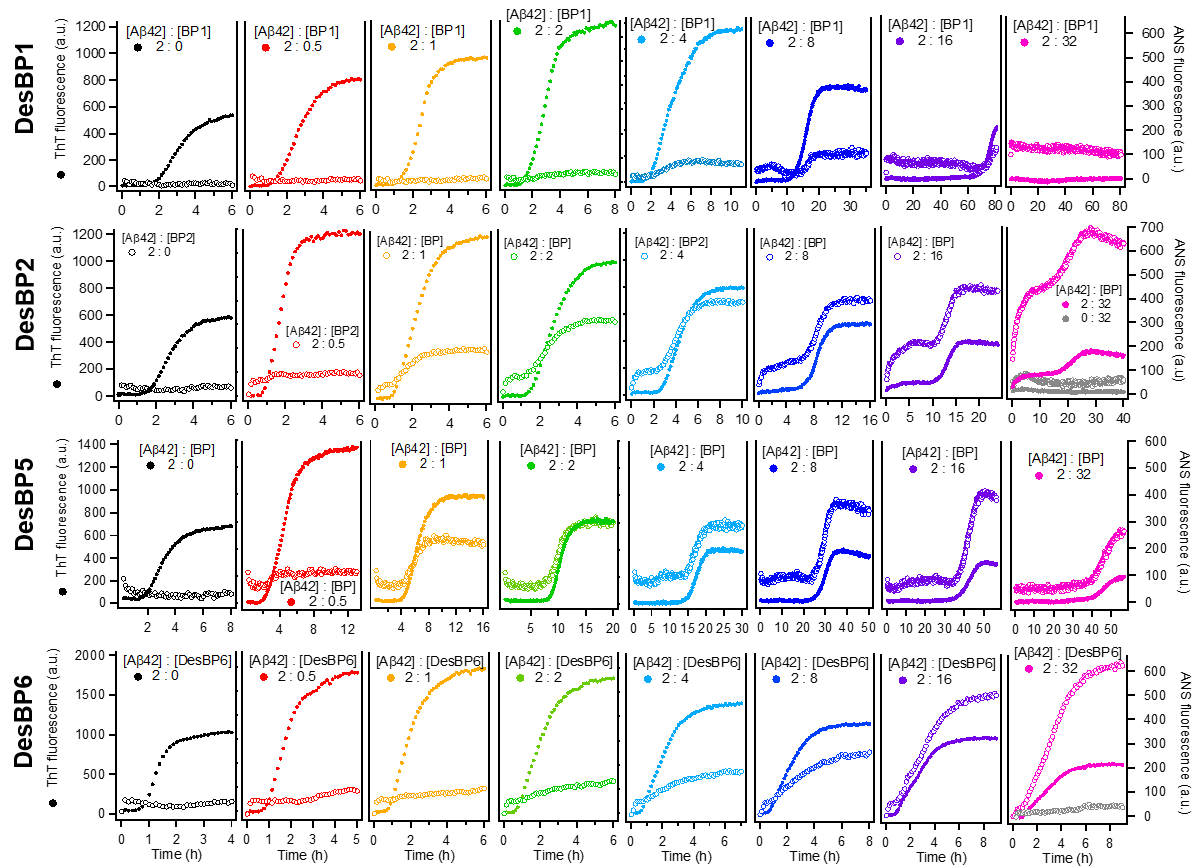
**

**Figure S4. Kinetic profile of ThT and ANS fluorescence of Aβ42 aggregation at various concentration of DesBPs.** ThT (closed circles) and ANS (opened circles) kinetic profiles of Aβ42 aggregation under quiescent conditions at a concentration of 2 μM in the absence or in the presence of various concentrations (0.5-32 μM) of DesBP1, DesBP2, DesBP5 and DesBP6 (represented by different colors). All experiments were performed in triplicate.

**
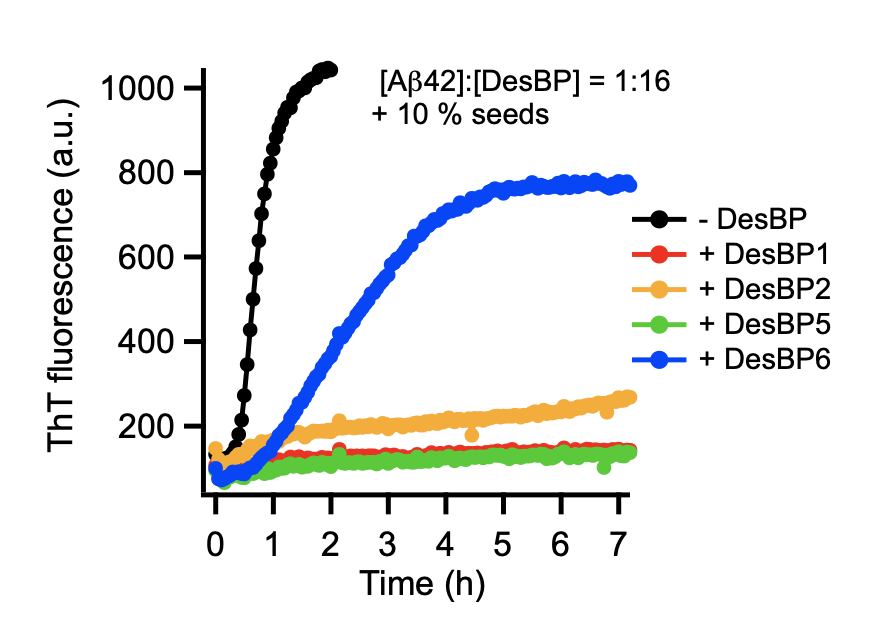
**

**Figure S5. Seeded aggregation assay of Aβ42 in the presence of high molar equivalents of DesBPs.** The aggregates formed in the presence of the DesBPs, with the exception of the case of DesBP6, did not show seeding ability indicating that they do not have a fibrillar nature. Experiments were performed in triplicate.

**
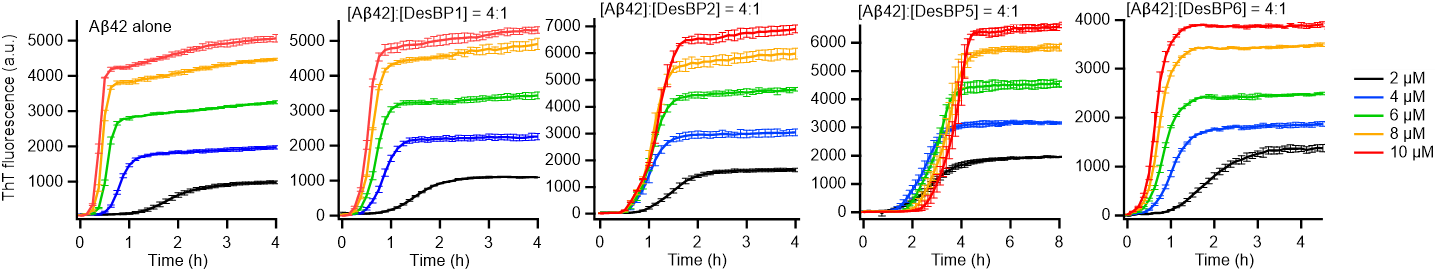
**

**Figure S6. Kinetics of Aβ42 aggregation in the presence of 0.25 molar equivalents DesBPs.** We report the results for increasing concentrations of Aβ42, from 2 to 10 μM. All experiments were performed in triplicate.
